# Combined Periodontal, Orthodontic, and Prosthetic Treatment in an Adult Patient

**DOI:** 10.1155/2015/716462

**Published:** 2015-10-26

**Authors:** Claudio Vinicius Sabatoski, Regis Claret Bueno, Ariel Adriano Reyes Pacheco, Matheus Melo Pithon, Orlando Motohiro Tanaka

**Affiliations:** ^1^Graduate Dentistry Program in Orthodontics, School of Health and Biosciences, Pontifícia Universidade Católica do Paraná, Curitiba, Brazil; ^2^Private Practice in Periodontics, Brazil; ^3^Southwest Bahia State University (UESB), Brazil; ^4^Brazilian Board of Orthodontics and Dentofacial Orthopedics, Brazil

## Abstract

A 41-year-old man had a significant loss of bone and supporting tissues with pathologic migration of several teeth and several missing teeth. He was treated with an interdisciplinary therapeutic protocol that included nonsurgical periodontal therapy based on strict control of supragingival plaque, subgingival periodontal therapy, orthodontic and endodontic treatment, and replacement of restorations. The orthodontic therapy was performed in a severely reduced bone support and the presence of pathological tooth migration after periodontal disease control. The interdisciplinary treatment protocol was the key to achieve a significant improvement in his facial and dental esthetics, masticatory function, and quality of life.

## 1. Introduction

The benefits of orthodontic treatment in adult patients have often been questioned because of the forces applied to the periodontal ligament and surrounding tissues. Orthodontic therapy in adults is challenging because of the interdisciplinary knowledge that is required. These patients need to be recognized before starting treatment to avoid exacerbation of the problem, and they should be informed of the periodontal risks of the orthodontic therapy [1–4].

Some risk factors associated with periodontal disease and gingival lesions are smoking, history of periodontal disease within the family, use of overdentures, hormonal, systemic diseases, nutritional deficiencies, stress, poor oral hygiene, allergic reactions, and occlusal trauma. Periodontitis is usually asymptomatic and if it is not treated it can lead to tooth loss [2–9]. Periodontal treatment must be performed before orthodontic treatment to restore and maintain the health of the supporting tissues [3, 6, 8, 10, 11].

The aim of the present paper was to describe the orthodontic treatment of an adult patient with chronic periodontal disease who presented with significant bone loss, pathologic tooth migration, and several missing teeth. Our goal was to provide an adequate esthetic and functional reconstruction of the occlusion while restoring his periodontal health.

## 2. Diagnosis and Etiology

**Figure 1:**
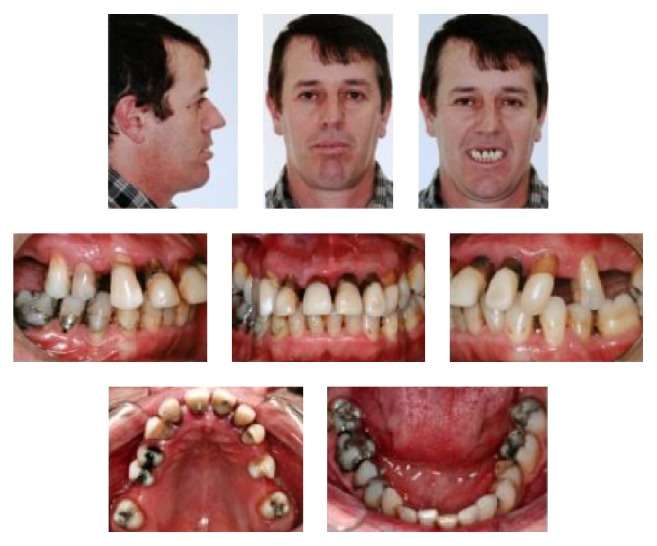
Pretreatment facial and intraoral photographs.

A male patient, aged 41 years and 3 months, was referred to our orthodontic office by his periodontist. His chief complaint was displaced maxillary anterior teeth, and he wanted to improve his facial esthetics (Figure 1). According to his periodontist, the patient was diagnosed with advanced periodontal disease with generalized bone loss. The periodontal treatment consisted of scaling, root planning, and oral hygiene instructions (Figures 3(a)–3(f)). The therapy took 6 months, and a 6-month observation period was followed to monitor patient cooperation and the stability of the periodontal results (Figures 3(g)–3(i)). After the periodontal disease control stage was completed, the possibility of orthodontic treatment was discussed.

In the facial photographs, the patient showed an impaired labial seal and excessive lower lip protrusion. Intraoral photographs and dental casts showed severe loss of periodontal support and pathological migration of maxillary anterior teeth with large gaps between the incisors. He had a canine Class I relationship on the left side and Class II on the right side, an overjet of 7.0 mm, and an overbite of 5.0 mm. The maxillary first molars and the second left premolar were absent, and the second molars inclined mesially. The mandibular midline deviated 2.5 mm to the right and the anterior teeth were extruded and slightly crowded (Figure 1).

**Figure 2:**
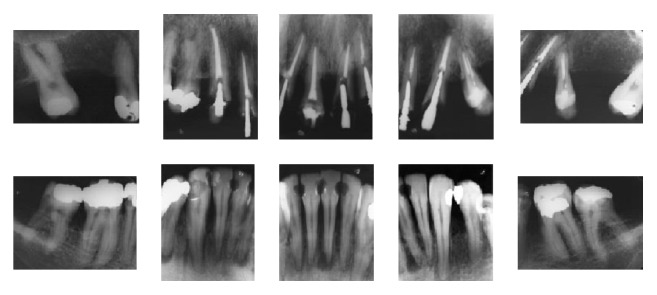
Pretreatment periapical radiographs.

The periapical radiographs (Figure 2) revealed severe bone loss in both the maxillary and mandibular arches. The apical radiolucent areas suggested periapical lesions of the mandibular left second premolar, mandibular left central incisor, and mandibular right first premolar.

### 2.1. Initial Treatment Objectives and Plan

The treatment objectives were to (1) align and level the teeth, (2) close the spaces and reduce the protrusion of the maxillary anterior teeth, (3) achieve an ideal overjet and overbite, (4) maintain a right Class II canine relationship and the mandibular midline deviation, (5) correct the mesial inclination of the maxillary second molar, (6) open the spaces to replace the missing maxillary first molars and left second bicuspid, (7) achieve a stable occlusion, and (8) improve the facial profile.

### 2.2. Treatment Alternatives

Two treatment choices were considered for this patient. Orthodontic and prosthodontic treatment, in conjunction with regular periodontal control, were presented to the patient. The first option was to align and level only the mandibular teeth with an orthodontic appliance, extract all the maxillary teeth, and place a complete denture supported by implants. This option was not conservative but required a relatively short treatment time. The second choice was to perform orthodontic treatment on both arches, intruding and uprighting the maxillary anterior teeth. The maxillary second molars were uprighted to place implants and crowns in the area of the maxillary first molars and in the area of the left second bicuspid. The mandibular teeth were aligned and leveled to achieve a stable occlusion.

### 2.3. Treatment Progress

**Figure 3:**
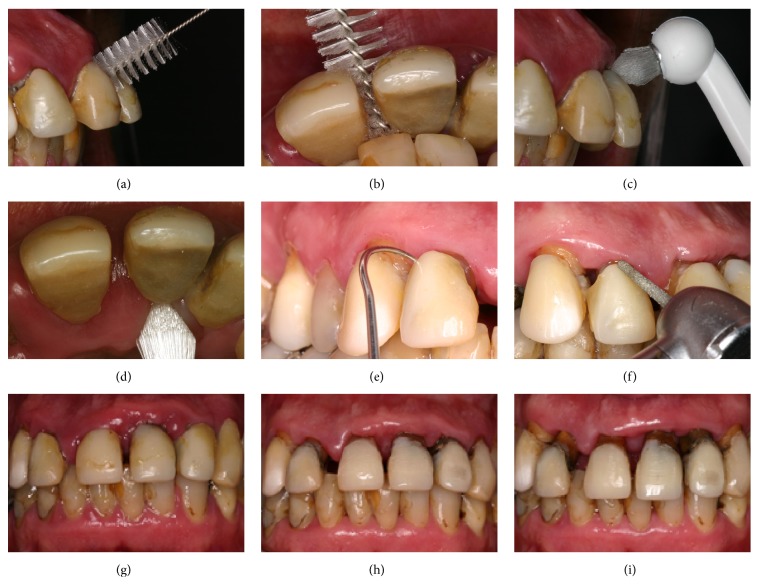
Periodontal treatment.

Before starting the orthodontic treatment, the patient was referred for endodontic and restorative procedures. Additionally, a 3-month periodontal recall schedule was established throughout the course of orthodontic therapy to emphasize oral hygiene instructions and periodontal disease control (Figure 3).

Treatment was initiated using a standard edgewise appliance with a 0.022 × 0.028 in slot. Due to the severely reduced periodontal support, light forces with good control of tooth movement were applied. In the maxillary arch, a sequence of archwires was used to perform alignment and leveling. We began with 0.012 in and 0.014 in NiTi, followed by 0.016 in, 0.018 in, 0.020 in, and 0.019 × 0.025 in ones.

**Figure 4:**
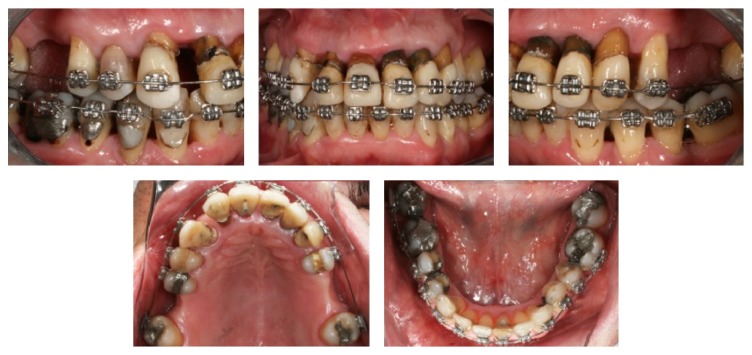
Treatment progress.

Retraction of the anterior maxillary teeth was performed with a 0.019 × 0.025 in stainless steel archwire with closing loops supported by Class II elastics. The force was verified and adjustments of the archwire were performed monthly. This procedure contributed to torque control and bodily movement of the maxillary anterior teeth. Anterior retraction was gradually performed over 8 months. In the mandibular arch, the same sequence of archwires was used to perform alignment and leveling, and the anterior teeth were stripped to eliminate crowding (Figure 4).

**Figure 5:**
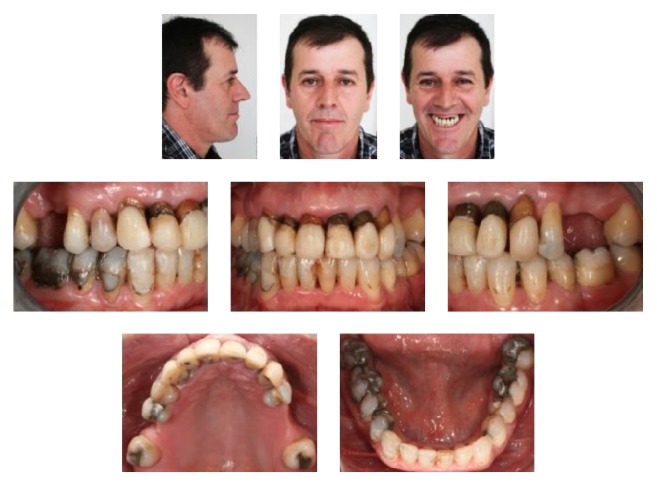
Posttreatment facial photographs and intraoral photographs.

The active treatment time was 30 months (Figure 5). After appliance removal, a removable Hawley-type retainer was placed in the maxillary arch, and a mandibular canine-to-canine lingual retainer was bonded. The patient was referred for restorative and prosthodontic treatment and was under a periodontal recall schedule every 3 to 6 months.

### 2.4. Treatment Results

The pathologically migrated maxillary anterior teeth were intruded and uprighted. Dental protrusion was reduced, the lips became competent, and his profile significantly improved. The posttreatment intraoral photographs showed a normal* overjet* and* overbite*. A Class I canine relationship on the left side and a mild Class II relationship on the right side were obtained. The mandibular midline was not corrected, and a slight deviation remained. The maxillary second molars were uprighted and adequate space for the missing teeth was established. His periodontal conditions remained unchanged, and the probing failed to reveal bleeding or other signs of active disease. His smile did not show good esthetics at that point in the treatment, but it greatly improved his self-confidence (Figure 5).

**Figure 6:**
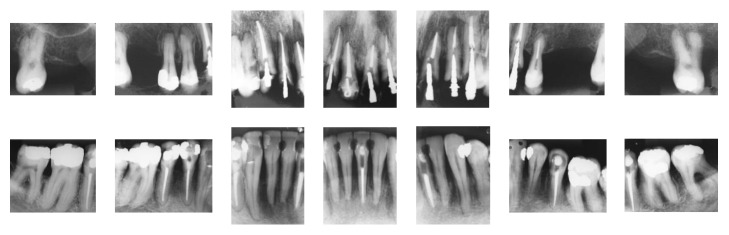
Posttreatment periapical radiographs.

The posttreatment periapical radiographs showed slight root resorption of the maxillary anterior teeth and the maintenance of bone levels (Figure 6).

**Figure 7:**
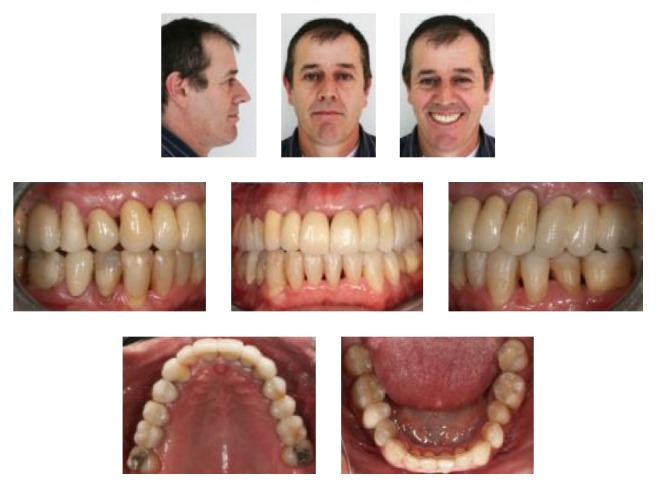
Posttreatment facial photographs and intraoral photographs 1 year and 11 months after debonding.

**Figure 8:**
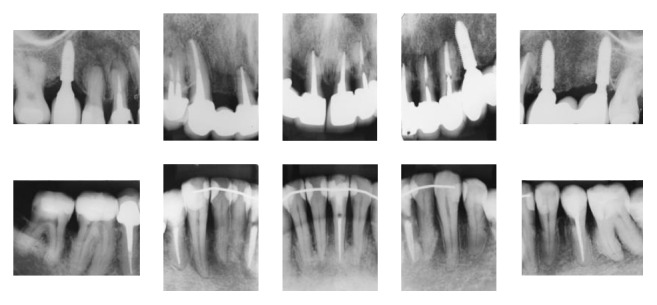
Posttreatment periapical radiographs 1 year and 11 months after debonding.

At the 1-year and 11-month follow-up, we observed the final results achieved after restorative and prosthodontic treatment. There was a great improvement in the esthetics of his smile. With the replacement of missing teeth, a stable occlusion was achieved and function was restored (Figure 7). The maxillary right lateral incisor had to be extracted due to a root fracture. The need for greater support guided the decision to extract the maxillary left first premolar and place an implant. The patient had maintained good periodontal health and bone levels remained consistent (Figure 8).

## 3. Discussion

Risk factors in adult patients must be identified prior to starting orthodontic treatment because aging increases the risk of periodontal problems. The ABO recommends at least one of the following procedures before beginning orthodontic treatment in these patients: (1) full mouth periodontal probing to detect gingival bleeding during probing, (2) written documentation certifying the periodontal treatment of the patient, (3) pretreatment panoramic with bitewings and periapical radiographs, and (4) full mouth periapical and bitewings radiographs [1, 4, 6].

The benefits of orthodontic treatment include improvement of dentofacial esthetics, osteogenic formation (this sometimes improves bony defects), and reestablishment of the occlusal plane, which eliminates occlusal trauma that together with periodontal disease leads to rapid destruction of periodontal tissues. Crowded malocclusions are more difficult to keep clean, so crowding may be a predisposing factor for periodontal disease. While orthodontic alignment would facilitate oral hygiene, there are no sufficient studies correlating malocclusion to periodontal disease [2, 3, 8, 12, 13]. Strong evidence questions the benefits of orthodontic treatment of periodontal patients claiming that the benefits of treatment do not exist and instead exacerbate the condition [12, 13]. In the present case report, the patient improved his periodontal health, dentofacial esthetics, and masticatory functions from the orthodontic treatment. The spaces from missing teeth were properly distributed for adequate prosthetic rehabilitation.

At the beginning of the treatment, the patient had flared incisors, difficulty sealing his mouth with his lips, rotations, hypereruption, and diastemas that all resulted from the pathologic migration, which lead to a relapse of gingivitis [11]. Pathologically flared incisors often have palatal pockets, so the retraction of these teeth must have an intrusion component to improve insertion. Retraction movements have an extrusive tendency [2, 3, 5], so in this case closing loops were inserted in the archwire to obtain vertical control of the retraction, avoiding an increase in the* overbite* to improve the insertion of the periodontal ligament. Activations were performed monthly and torque readout to control the anterior teeth was performed at each appointment during the retraction phase to obtaining bodily tooth movement while avoiding buccolingual movements, which are known to be riskier and potentially harmful and undesirable in a case like this [4, 6, 12, 14].

Controlled orthodontic treatment, when performed after periodontal stability has been achieved, does not appear to increase or activate the disease [2, 8]; however, the patient's full collaboration is needed. In this case report, the patient attended his periodontal appointments before starting orthodontic treatment and every three months during treatment. In addition, if a systemic disease is present, it must be controlled. Smoking should also be ceased, and occlusal stress must be reduced [3, 4, 6].

Due to the poor periodontal status, continuous light forces to move the teeth and minimize occlusal trauma were used because the center of resistance is located more apically due to bone loss. This would lead to a more physiological frontal bone resorption and, therefore, to a quicker tooth movement. High forces could potentially interfere with the remodeling process [2, 5, 8, 15, 16]. This must be specially recalled because the aplastic cortical bone of adults normally shows delayed bone formation and regeneration [3]. At the beginning of the alignment, small diameter NiTi wires were chosen. We then moved to stainless steel wires that were initially placed passively and were then slowly rectified at each appointment. This provided better control and avoided the release of high intensity forces. In this case, molar bands were avoided and the use of bonded orthodontic molar tubes was preferred because if the band is not properly adapted, it could harm the subgingival supporting tissues, leading to inflammation and subsequent alveolar bone loss [4, 11, 12, 17]. The borders of the prosthetic crowns were also relieved.

At the beginning of the treatment, we were careful and concerned about bacterial colonization of the brackets and ligatures. Some authors recommend the use of self-ligated brackets instead of conventional brackets, claiming that the elastomeric rings are more prone to bleeding, and SLB's accumulate less plaque than conventional brackets, thus improving oral hygiene [18, 19]. We opted for conventional brackets with stainless steel ligatures because those findings are not supported by other authors [20]. In these patients, a long movement of the teeth over their biological limits should be avoided to prevent crestal alveolar bone loss. Intrusion and retraction movements toward the bone have a potential osteogenicity, which improves periodontal attachment [3].

Permanent retention is advisable, although relapse seems to not present a problem due to displacement of the periodontal fibers during the surgical phases of periodontal treatment [3]. However, after removing the appliance, fixed lingual retainers were bonded for retention in the mandible, and a Hawley maxillary retainer was also used. Removable aligners are a good alternative for retention.

Orthodontic treatment of a periodontal patient with pathologic tooth migration is effective if there is multidisciplinary cooperation. Mutual aggravation of periodontitis and occlusal trauma can be prevented by treatment. The improvement of facial esthetics contributed to the self-confidence of an adult periodontal patient with pathologic tooth migration [16].

Orthodontic treatment of adult patients with periodontal disease must have periodontal disease control before, during, and after orthodontic treatment. But the main factors to be addressed in periodontal therapy are the types and virulence of the provocative organisms and not the ability of the host to resist the aggression [21].

Although the orthodontic treatment was finished, the periodontist required the patient to be reviewed every 6 months to prevent reinfection and recurrence after the successful treatment. The patient knew that uncontrolled follow-up plaque control could contribute to periodontal disease with the occurrence of inflammation, with bone dehiscences, fenestrations, and chronic inflammation of the gingival tissues and the reactivation of the previously controlled periodontal disease. The patient should continue a program of regular follow-up visits to the periodontist and the orthodontist, and meticulous hygiene must be maintained throughout life. In general, in the absence of active periodontal disease and with good oral hygiene the maintenance of good periodontal health and bone levels might remain consistent.

## 4. Conclusion

The favorable results achieved in this case report show that it is possible to complete orthodontic treatment in a patient with severely reduced bone support and pathological tooth migration if good periodontal disease control is achieved. The interdisciplinary treatment protocol is the key to significantly improve the restoration of function, esthetics, and quality of life in adult patients. The patient was pleased with the result provided by the various dental specialties.
